# Pyogenic Liver Abscess Following Therapeutic Endoscopic Retrograde Cholangiopancreatography (ERCP) and Robot-Assisted Cholecystectomy: A Case Report

**DOI:** 10.7759/cureus.114548

**Published:** 2026-08-14

**Authors:** Sara Sadeghi, Maureen Okafor, Eugene Choe, Jarin Prasa, Michael Bernstein

**Affiliations:** 1 Medicine, NYC Health + Hospitals South Brooklyn Health, Brooklyn, USA; 2 Medicine, St. George's University School of Medicine, St. George's, GRD; 3 Gastroenterology, NYC Health + Hospitals South Brooklyn Health, Brooklyn, USA

**Keywords:** biliary sphincterotomy, endoscopic retrograde cholangiopancreatography (ercp), gi endoscopy, klebsiella liver abscess, percutaneous abscess drainage, pyogenic liver abscess due to klebsiella pneumoniae, pyogenic liver abscesses, robot-assisted cholecystectomy

## Abstract

Pyogenic liver abscess is a potentially life-threatening condition primarily caused by bacterial spread from the gastrointestinal or biliary tract to the liver. As endoscopic retrograde cholangiopancreatography (ERCP) with biliary sphincterotomy becomes more common in the treatment of choledocholithiasis, the risk of developing pyogenic liver abscess increases, with *Klebsiella pneumoniae* continuing to emerge as a more commonly identified pathogen in North America. Our case describes this unique presentation of a 54-year-old female diagnosed with *Klebsiella* liver abscess (KLA) following multiple diagnostic and therapeutic interventions: ERCP, biliary sphincterotomy with stent placement, and robot-assisted cholecystectomy. Prophylactic antibiotics, meticulous aseptic techniques during procedures, and close postoperative monitoring are key strategies that may mitigate the risk of KLA. In addition, clinical vigilance for potential bacterial colonization in these high-risk patients may further reduce the incidence of such complications. Importantly, this study highlights the gaps in science and the need for further investigation of endoscopic interventions as potential underlying risk factors associated with pyogenic liver abscess, particularly KLA.

## Introduction

Pyogenic liver abscess is a potentially life-threatening condition with a high mortality rate ranging from 10% to 40% [1]. This serious infectious condition is primarily caused by bacterial spread from the gastrointestinal or biliary tract to the liver, resulting in either hepatic microabscesses or macroabscesses [2]. In patients undergoing hepatobiliary interventions, disruption of the normal biliary-enteric barrier is known to increase the risk of retrograde bacterial colonization, cholestasis, stent-related biliary injury, possible biofilm formation over indwelling implants, and immune suppression [3,4]. Endoscopic retrograde cholangiopancreatography (ERCP) with biliary sphincterotomy is a common procedure for treating choledocholithiasis. A cohort study of the largest hospital system in Taiwan demonstrated that patients undergoing ERCP with endoscopic sphincterotomy have a significantly higher cumulative incidence of pyogenic liver abscess compared to those receiving other ERCP procedures (five-year cumulative incidence 2.4% vs. 1.7%) [5]. Further, prior cholecystectomy has been reported in a few cases of pyogenic liver abscess. Although one retrospective study reported 40% of patients to have either had biliary tract disease or biliary intervention like cholecystectomy or ERCP [6], risk associations are less understood.

Recent studies have reported that in North America, *Klebsiella pneumoniae* is emerging as a more commonly identified pathogen of pyogenic liver abscess compared to *Escherichia coli* [7]. Typically, pyogenic liver abscesses are known to develop via several mechanisms, including (1) direct spread from biliary tract infections, (2) portal venous spread from intra-abdominal infections, (3) systemic hematogenous seeding, (4) from trauma or penetrating wounds, (5) from ingested foreign bodies, or (6) cryptogenically [2,8-10]. These abscesses occur most commonly in the right hepatic lobe due to its larger size and blood supply [11].

In this case, we report this unique presentation of a rare yet serious occurrence of *Klebsiella* liver abscess (KLA) following multiple diagnostic and therapeutic interventions - ERCP, biliary sphincterotomy with stent placement, and robot-assisted cholecystectomy - a potential risk factor for KLA [7,12,13].

## Case presentation

**Figure 1:**
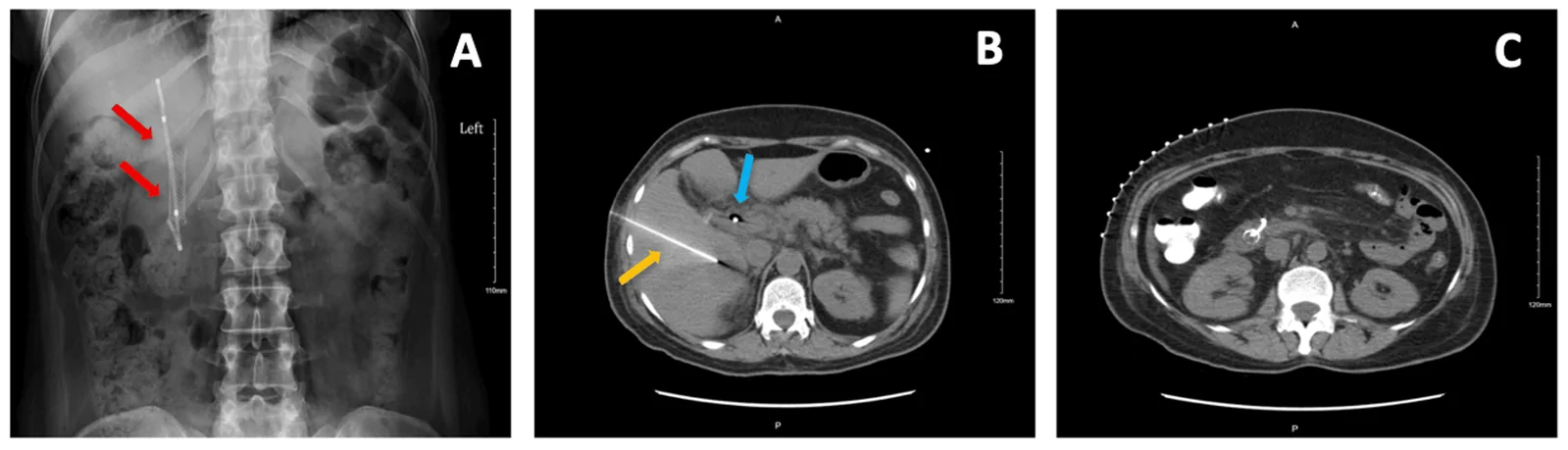
Post-cholecystectomy common bile duct (CBD) and internal biliary stents (red arrows) visualized on abdominal X-ray (A). Under CT guidance, a 21-gauge micropuncture needle (yellow arrow) advanced into the hepatic abscess (B). An ill-defined heterogeneous lesion in the R hepatic lobe with pneumobilia (blue arrow) and a CBD stent is noted (B, C).

The patient is a 54-year-old female with no chronic medical condition who presented to the Gastroenterology clinic for a follow-up visit with complaints of fever, chills, nausea, and right upper quadrant abdominal pain for four days. About four weeks prior to her visit, she had been evaluated for acute cholecystitis and choledocholithiasis, for which she underwent a diagnostic ERCP and biliary sphincterotomy with stent placement in the common bile duct (CBD) (Figures 1A-C). The scope was advanced to a normal major papilla, and a short-nosed traction sphincterotome was passed over a 0.035-inch × 260 straight guidewire for deep bile duct cannulation, revealing CBD dilatation. A biliary sphincterotomy was made using a braided sphincterotome, and the biliary tree swept with an 8.5 mm × 4 cm balloon dilator starting at the bifurcation. Following removal of sludge and stone, a 10 mm × 4 cm fully covered metal stent was placed in the CBD, and bile flow was then appreciated. Then, a 7 Fr × 5 cm plastic stent with a full external and internal pigtail was properly positioned in the CBD through the first stent to prevent stent obstruction. Preoperatively, intravenous cefoxitin was administered prophylactically, and post-procedure, rectal indomethacin was administered to prevent post-ERCP pancreatitis. This procedure was followed a day later with a robot-assisted cholecystectomy. An intact gallbladder was taken down from the liver bed and extracted via an endoscopic retrieval basket. No bile leak was noted from the cystic duct stump. Both procedures were completed with no immediate complications. Following both procedures, her hospital stay was unremarkable. She was clinically stable, tolerating diet well, and was safely discharged home.

In this current encounter, the patient was referred to the ED with concerns for an infectious etiology. Although an initial leukocyte count was normal (9.4 K/mcL), laboratory findings were remarkable for an elevated high-sensitivity C-reactive protein (249 mg/L), mildly elevated alanine transaminase (50 U/L) and aspartate transaminase (50 U/L), hypokalemia (3.4 mmol/L), and mildly elevated lactate (1.5 mmol/L) and INR (1.37) (Table 1).

**Table 1:** Laboratory Findings on Hospital Presentation and on Discharge. Abbreviations: AST: aspartate transaminase; ALT: alanine transaminase; ALK: alkaline phosphatase; INR: international normalized ratio; CRP: C-reactive protein; BUN: blood urea nitrogen.

Laboratory Test	Initial Encounter	On Discharge	Reference Ranges
White blood cells	9.57	5.22	4.7–10.3 K/mcL
Hemoglobin	11.4	11.5	12.0–16.0 g/dL
Platelets	137	339	150–450 × 10³/mcL
AST	50	37	≤35 U/L
ALT	50	26	≤35 U/L
ALK	73	91	35–104 U/L
INR	1.37	1.01	0.91–1.10
Total bilirubin	0.8	0.3	<1.2 mg/dL
Direct bilirubin	0.2	-	0.0–0.3 mg/dL
Indirect bilirubin	0.6	-	0.0–0.8 mg/dL
Lipase	14	-	13–60 U/L
Lactate	1.5	-	0.5–2.2 mmol/L
High-sensitivity CRP	249	3.7	≤5.0 mg/L
Sodium	137	140	136–145 mmol/L
Potassium	3.4	3.9	3.5–5.1 mmol/L
BUN	9	10	6–20 mg/dL
Creatinine	0.5	0.5	0.5–0.9 mg/dL
Bicarbonate	20	25	22–29 mmol/L
Glucose	74	90	70–100 mg/dL

**Figure 2:**
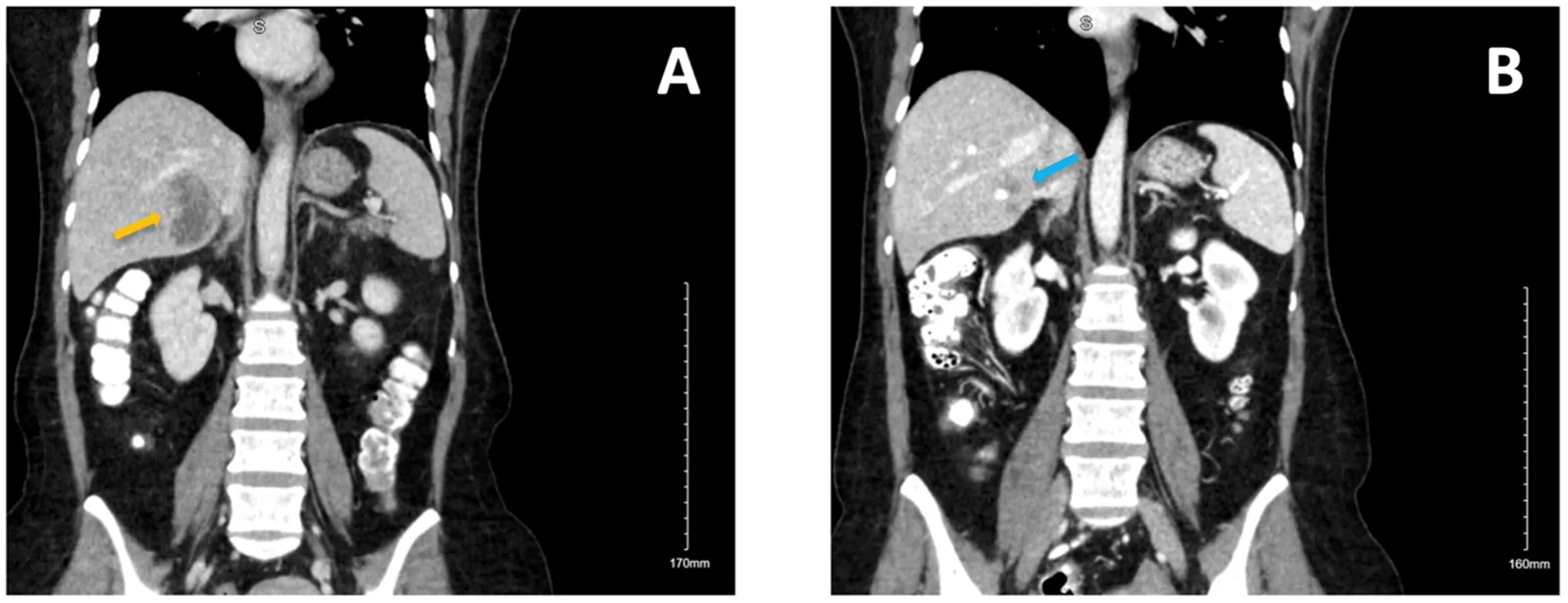
Coronal CT views of a complex, heterogenous lesion (yellow arrow) in the right hepatic lobe, initially measuring 5.2 x 2.8 x 4.2 cm (A). Interval decreases in size (blue arrow) are noted on subsequent imaging, down to 2.2 x 2.2 x 1.2 cm (B).

Computed tomography (CT) of her abdomen and pelvis revealed a posteriorly located 5.2 x 2.8 x 4.2 cm complex lesion in the right hepatic lobe (Figure 2A). She was then started on a broad-spectrum carbapenem antibiotic. Within 10 hours of admission, she developed septic shock with hemodynamic instability requiring vasopressor support and immediate medical intensive care.

A CT-guided percutaneous drainage catheter was promptly inserted (Figure 1B), which yielded serosanguinous purulent fluid and was maintained in situ for further drainage. Abscess fluid culture returned with growth of *Klebsiella pneumoniae*. *Echinococcus*, *Entamoeba* histolytica serologies, HIV, tuberculosis Quantiferon, and urine and blood culture tests were negative. A diagnosis of pyogenic liver abscess was made. With source control completed and the patient’s symptoms abated, she showed prompt clinical improvement with no evidence of metastatic infection. Her antibiotic therapy was de-escalated to cefazolin based on the susceptibility findings from culture results and local antibiograms.

One week later, a repeat abdominopelvic CT scan confirmed a decrease in the liver abscess size to 2.8 x 2.6 x 2.6 cm. With a minimum amount of drainage via the drainage catheter, it was subsequently removed 13 days after it was placed. Laboratory findings returned to normal ranges, and the patient was discharged home. She subsequently completed a five-week total course of outpatient antibiotic therapy. An outpatient clinic follow-up with an abdominopelvic CT was scheduled (Figure 2B), showing a continued interval decrease in the size of the hepatic lesion.

## Discussion

Given the anatomical connection between the biliary system and gastrointestinal tract, there exists a potential to transfer microorganisms from the bowel lumen to the upper biliary system during an ERCP procedure, which subsequently could lead to the development of a pyogenic liver abscess. There are relatively rare but reported instances of this adverse sequela, and not all patients undergoing ERCP with or without sphincterotomy develop such a complication [12].

Of note, previous studies have described *K. pneumoniae* liver abscesses to be cryptogenic, with patients more often presenting with vague symptoms [7,14]. In this index case, our patient also presented with non-specific symptoms of fever, chills, nausea, and abdominal pain. Furthermore, she developed significantly elevated high-sensitivity C-reactive protein despite a normal white blood cell count. Li et al. reported similar findings in 206 patients with pyogenic liver abscess, in which there was no statistically significant difference in white blood cell count among patients with sepsis compared to those without, but a significant elevation in C-reactive protein for patients with sepsis versus those without [15]. These features highlight a unique clinical pattern of early sepsis with increased acute inflammatory markers preceding leukocytosis in patients with pyogenic liver abscess.

The occurrence of KLA following ERCP with biliary sphincterotomy, as presented in this case, also underscores the importance of recognizing *Klebsiella pneumoniae* as a significant etiologic agent in pyogenic liver abscess. While *Escherichia coli* is commonly implicated in post-ERCP liver abscess, identifying *Klebsiella* sp. in this context may indicate a serious complication. Wu et al. reported that the incidence of pyogenic liver abscess following ERCP for choledocholithiasis was higher in an endoscopic sphincterotomy group compared to a non-sphincterotomy group, with *Klebsiella pneumoniae* being a notable pathogen [5].

No studies have explored dose-related associations between endoscopic procedures and pyogenic liver abscess, but some retrospective studies suggest that a prior history of biliary endoscopic procedures and multiple comorbidities are characteristic of high-risk patients [16,13]. As with this case, antibiotic therapy and radiologic drainage remain the mainstay treatment of pyogenic liver abscess [17]. Alternatively, surgical drainage has been found beneficial in patients with large or multiloculated hepatic abscesses [18]. Nevertheless, preventive measures following ERCP, biliary sphincterotomy, or robot-assisted surgery may be valuable, especially given the increased risk of disease associated with interventional procedures [13].

Prophylactic antibiotics, meticulous aseptic techniques during procedures, and close postoperative monitoring remain key strategies necessary to mitigate the risk of KLA [19,20]. In addition, clinical vigilance for potential bacterial colonization in these high-risk patients [21] may further reduce the incidence of such complications. Ultimately, although no evidence exists for routine screening of high-risk patients, it may be useful to develop standardized guidelines for the prevention and early detection of pyogenic liver abscess in patients undergoing these endoscopic procedures.

## Conclusions

In summary, there is very limited to no understanding of the risk of KLA in patients who undergo several endoscopic and/or robot-assisted interventional procedures, or whether this risk is exponential in relation to the number of procedures conducted. Although this case may highlight a need to explore the value of clinical vigilance and tailored antimicrobial strategies to effectively and preemptively manage such infections, it more importantly reveals the gaps in science and the need for further investigation of endoscopic interventions as underlying risk factors associated with pyogenic liver abscess, particularly KLA.
